# Trajectory of chemical cocktail-induced neutrophil reprogramming

**DOI:** 10.1186/s13045-020-01008-8

**Published:** 2020-12-10

**Authors:** Yi Zhou, Chuijin Wei, Shumin Xiong, Liaoliao Dong, Zhu Chen, Sai-Juan Chen, Lin Cheng

**Affiliations:** Shanghai Institute of Hematology, State Key Laboratory of Medical Genomics, National Research Center for Translational Medicine at Shanghai, Ruijin Hospital Affiliated to Shanghai Jiao Tong University School of Medicine, Shanghai, 200025 China

**Keywords:** CellTagging, Single-cell RNA sequence, Neutrophils, Hematopoietic reprogramming, Trajectory

## Abstract

Hematopoietic reprogramming holds great promise for generating functional target cells and provides new angle for understanding hematopoiesis. We reported before for the first time that diverse differentiated hematopoietic cell lineages could be reprogrammed back into hematopoietic stem/progenitor cell-like cells by chemical cocktail. However, the exact cell types of induced cells and reprogramming trajectory remain elusive. Here, based on genetic tracing method CellTagging and single-cell RNA sequencing, it is found that neutrophils could be reprogrammed into multipotent progenitors, which acquire multi-differentiation potential both in vitro and in vivo, including into lymphoid cells. Construction of trajectory map of the reprogramming procession shows that mature neutrophils follow their canonical developmental route reversely into immature ones, premature ones, granulocyte/monocyte progenitors, common myeloid progenitors, and then the terminal cells, which is stage by stage or skips intermediate stages. Collectively, this study provides a precise dissection of hematopoietic reprogramming procession and sheds light on chemical cocktail-induction of hematopoietic stem cells.

To the editor,

Hematopoietic reprogramming against hematopoietic differentiation, from one type of differentiated hematopoietic cells transdifferentiating into other types or dedifferentiating into progenitors or stem cells, is considered as another paradigm in the field of hematology and stem cells [1, 2]. Modulators which enabled hematopoietic reprogramming have been mainly limited to transcription factors [3], which is same to the cell reprogramming to generate non-hematopoietic cells, such as pluripotent stem cells and solid tissue somatic cells [4]. However, virus-mediated exogenous gene expression is hardly to be translated into clinical application due to safety concerns, efficiency, etc. Small molecule chemical compounds with many advantages comparing with the above method have been considered as an alternative for manipulating cell reprogramming [5]. Many functional target cells have been induced via diverse chemical compounds-enabled cell reprogramming strategies in recent decade, although the precise reprogramming trajectories for each target cells are not fully elucidated.

We previously demonstrated for the first time that different lineages of differentiated hematopoietic cells could be reprogrammed back into hematopoietic stem/progenitor cell (HSPC)-like cells by a chemical cocktail [6]. This report is a proof of concept that chemical cocktail-induced hematopoietic cell reprogramming is practical, except for the exogenous gene-induction, which leaves the cellular mechanism of reprogramming trajectory unresolved. Genetic tracing combining with single-cell analysis is a cutting-edge technique and proven to be a powerful tool for analyzing cell reprogramming processions and redefining developmental processions [7], such as hematopoiesis. In this paper, we intend to dissect the cell conversion procession of the chemical cocktail-induced hematopoietic reprogramming based on CellTagging method and single-cell RNA sequence.

CellTagging technique contains 8 bp random nucleotides as tags that are heritable and transcriptable, which allows to capture every single-cell identity and evolving trajectory parallelly [8, 9]. To label the initial hematopoietic cells, we infected the mouse bone marrow-derived mononuclear cells with lentivirus expressing CellTags and green fluorescent protein (GFP). 32 h after infection and cell expansion in vitro, GFP positive cells were isolated via cell sorting. 3/4 of the cells were harvested as day 1 and the remaining cells were further cultured and treated with chemical cocktail containing 0.5 mM Valproic acid (VPA), 3 μM CHIR99021, and 1 μM Repsox for 7 days. Single-cell RNA sequence of these cells at both timepoints with 10 × Genomics, which included ~ 25,000 individual cells in total with ~ 2,000 median genes and ~ 20,000 mean confidently mapped reads per cell (Additional file 1: Figure S1A), showed that CellTags-labelled differentiated cells on day 1 included neutrophils, eosinophils, basophils, macrophages, T cells, and erythrocytes. Major cells on day 7 acquired HSPC program (Fig. 1a) with *Ly6e*, *Lmo2*, *Hoxa9*, *Runx1*, *Cd34*, *Gfi1*, *Egfl7*, *Myl10*, *Ctsg*, and *Prtn3* being activated (Additional file 1: Figure S1B). This result is consistent with our data reported before. Thus, the CellTags do not disturb the hematopoietic cell reprogramming efficiency. In these cells, insertion number of CellTags per cell was from 1 to 40. Average number was 2 and there was no difference between day 1 and day 7 (Additional file 1: Figure S1C). 5,137 cells from two timepoints were tagged with the same CellTags (Additional file 1:Figure S1D). According to these CellTags, it was found that 43% of the induced cells acquiring HSPC program were derived from neutrophil lineages (All CellTags inserted in neutrophils are shown in Additional file 2: Table S1), 30% from eosinophil lineages, 15% from macrophage lineages, 6% from basophil lineages, 3% from erythrocyte lineages, and 3% from T cells (Fig. 1b). Further analysis showed that the induced cells with HSPC program were heterogeneous and could be clustered into several subpopulations (Additional file 1: Figure S1E). All together, these data not only validate our previous report further with CellTagging approach as genetic tracing, but also demonstrate that HSPC-like cells could be generated from neutrophil lineages by the chemical cocktail-induced reprogramming with the highest contribution due to their cell number advantage among all the initial differentiated hematopoietic cells.

**Fig. 1:**
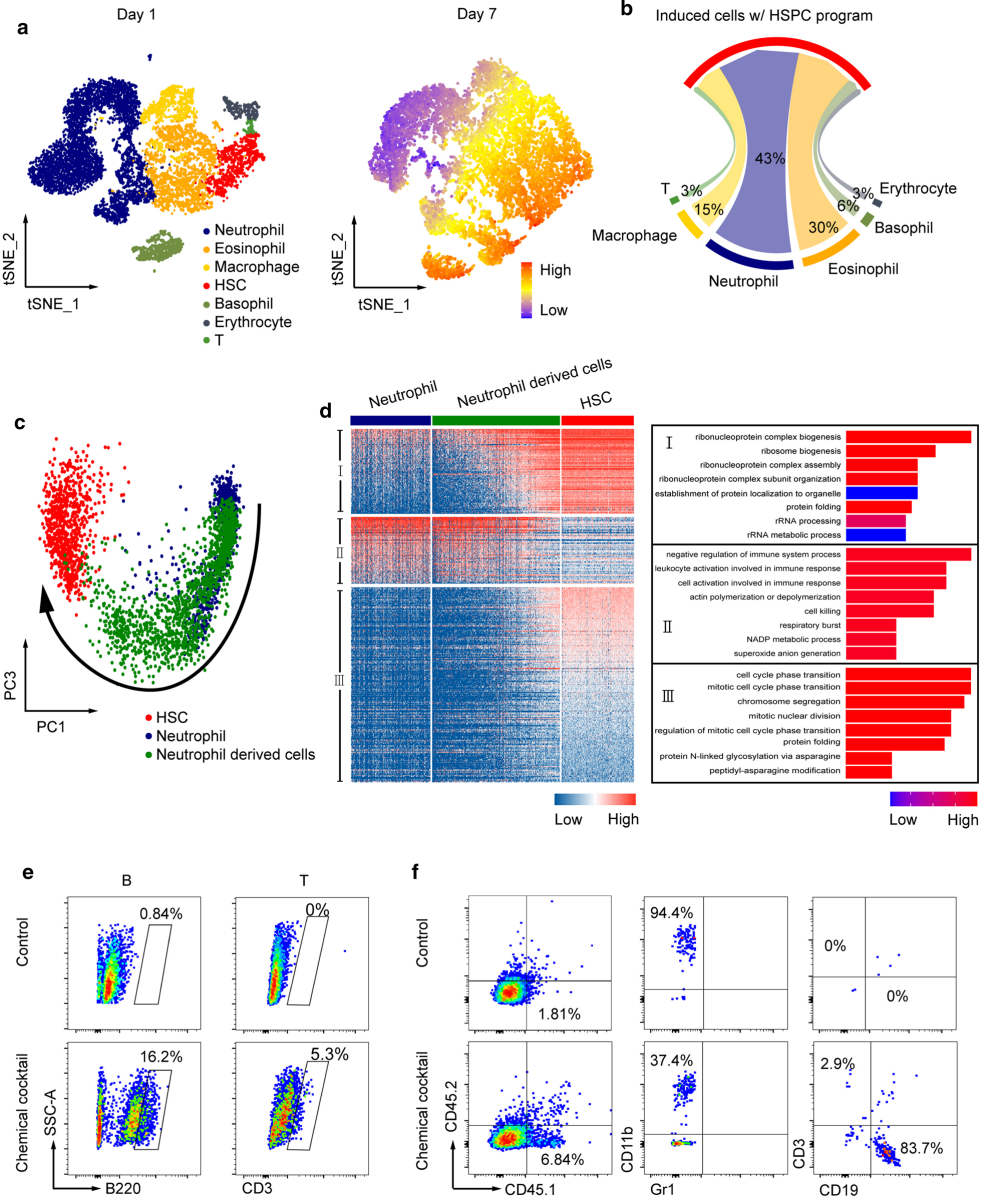
Neutrophils labelled by CellTags were Reprogrammed into MPP. **a** t-SNE visualization of 12,521 cells labelled by CellTags on day 1 (left) and 13,547 CellTags-labelled cells with HSPC program on day 7. “High” and “Low” indicate the mean expression levels of HSPC gene sets. **b** Sankey diagram showed the reprogramming efficiency from initial hematopoietic cell lineages into induced cells with HSPC program. **c** PCA analysis of primary HSC, initial neutrophils and the neutrophil derived cells revealing cell fate transition of chemical cocktail-induced reprogramming. **d** Clustering heatmap of 389 top DEGs of the cells shown in (**c**) (left) and GO analysis of the three gene sets (right). **e** Neutrophil-derived cells were cultured on feeder cells then analyzed by FACS for lymphoid cell markers. **f** Neutrophil-derived cells (CD45.1) were transplanted into sublethal irradiated CD45.2 mice. Donor cells in recipient peripheral blood were monitored by FACS analysis

It was apparent that neutrophils transited toward hematopoietic stem cells (HSC) with the chemical cocktail induction by principal component analysis. A few neutrophil derived cells, namely neutrophils treated with chemical cocktail, were clustered even together with primary HSC (Fig. 1c). It was supported by hierarchical clustering analysis of these three cell groups, which also showed that expression of neutrophil specific genes, such as *S100a6*, *S100a11*, *Ly6c2*, and *Samsn1*, was gradually suppressed as the HSPC genes, such as *Prtn3*, *Runx1*, *Cd34*, *ly6e*, and *Lmo2*, were partially or completely activated (Additional file 3: Table S2). There were three coordinate gene sets clearly identified by analyzing the top signature genes. Gene ontology enrichment showed that gene set I was related with ribosome activity and rRNA processing. Gene set II was mainly about immune response related with myeloid cell function, especially neutrophils. Gene set III was about cell cycle phase transition and chromosome conformation change (Fig. 1d). Dot plot analysis verified the signature of corresponding phenotypes in each gene sets (Additional file 1: Figure S1F). Together, partial neutrophil derived cells by chemical cocktail induction are highly close to HSC according to transcriptional signature.

To verify the neutrophil-derived cell function, firstly, we cultured the cells on feeder cells OP9 or OP9-DL1, which have been used for promoting HSC differentiation into lymphoid cells [10, 11]. It was found that 16.2% cells differentiated from chemical cocktail-induced cells were B220 positive and 5.3% cells were CD3 positive (Fig. 1e), which are markers of B cells and T cells individually. Secondly, we transplanted the induced cells derived from CD45.1 neutrophils into sublethal irradiated CD45.2 mice. Two weeks after transplantation, higher percentage of CD45.1 positive cells in the peripheral blood of recipient mice was detected for the chemical cocktail-induced donor cells comparing with control one. Among those chimeric cells, 52.4% were CD19 positive and 1.81% were CD3 positive in the chemical cocktail group, standing for B cell and T cell generation in vivo, which were not monitored in control group (Fig. 1f). All these data demonstrated that the chemical cocktail-induced cells from neutrophils acquired the function of multi-differentiation potential into lymphoid cells both in vitro and in vivo, expect for myeloid cells and erythroid cells as we reported before.

Next, we intend to identify the neutrophil-derived cell types and their connection by the chemical cocktail induction. Primary hierarchical clustering analysis showed that the cells could be classified into seven groups, which included multipotent progenitors (MPP), granulocyte/monocyte progenitors (GMP), common myeloid progenitors (CMP), megakaryocyte/erythrocyte progenitors (MEP), preneutrophils, immature neutrophils, and mature neutrophils (Fig. 2a). A cell-fate conversion trajectory among those induced cells was constructed based on Scanpy-based algorithm [12] and partition-based graph abstraction algorithm [13] (Fig. 2b). From the trajectory map, it was found out that each preneutrophils, immature neutrophils, and mature neutrophils could be further divided into three subtypes of cell lineages. Generally, under the chemical cocktail induction, mature neutrophils were reprogrammed back into immature ones then into premature ones, which was complete reversion of neutrophil development procession. Unexpectedly, most of the induced preneutrophils were further converted directly into MPP, although a few of the cells were further induced into GMP first then into CMP then into MPP, still following the developmental route reversely. Interestingly, MEP were not only generated from induced CMP and MPP but also inducted directly from preneutrophils and immature neutrophils. Mature neutrophils could directly be reprogrammed into premature ones, skipping the immature stage. Analysis based on monocle algorithm validated the above result (Additional file 4: Figure S2A). Specific gene expression for each cell types is shown representatively in Fig. 2c. Mature neutrophils include *Itgam* and *Mmp9*, immature neutrophils contain *Gal* and *Gngt2*, preneutrophils have *Ctsg* and *Elane*, GMP include *Ifitm1* and *Gata2*, CMP contain *Ctsc* and *Prss34*, MPP have *Gfi1* and *Prtn2*, and MEP contain *Car1* and *Hba-a1*.

**Fig. 2:**
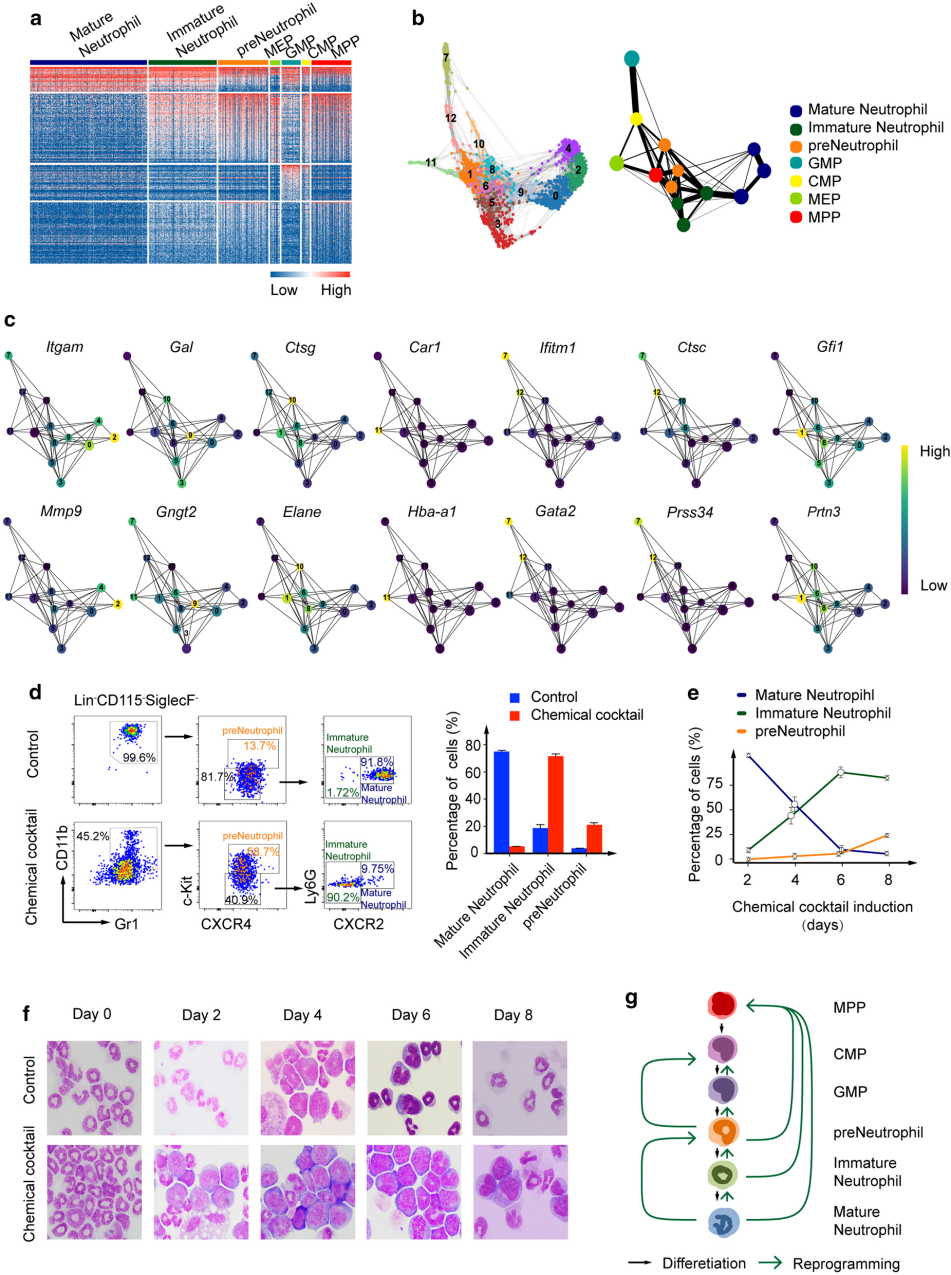
Construction of neutrophil reprogramming trajectory by the chemical cocktail induction. **a** Clustering heatmap of 1600 top DEGs of the neutrophil derived cells by chemical cocktail induction on day 7. **b** Topological map of neutrophil reprogramming. FA visualization of 1468 cells induced from neutrophils was identified by Leiden clustering algorithm in Scanpy. The relationship of clusters was calculated by PAGA algorithm (right). **c** Mean expression of specific genes for each cluster in PAGA layout. **d** FACS analysis of neutrophil markers with different maturation stages on day 8 after chemical cocktail induction (left). Statistical analysis of cell percentage (right). **e** Quantification of cell percentage of neutrophils with different maturation stages on different days after chemical cocktail induction. **f** Wright-Giemsa staining of cells on different days. **g** Schematic model of the main trajectory map of chemical cocktail-induced neutrophil reprogramming

Considering the total neutrophils on day 1 were also clustered into three subtypes, which made the above established converting procession from mature neutrophils into immature ones then into premature ones could not be wholly attributed to the chemical cocktail-enabled cell reprogramming. Alignment of CellTags of every cell from day 1 to day 7 demonstrated that mature neutrophils could be induced back into immature ones, premature ones, and other diverse progenitors step by step (Additional file 4: Figure S2B). Immature neutrophils were reprogrammed into premature ones, and progenitors and initial preneutrophils were converted into different progenitors. Among those induced cell types, CMP and MEP were generated at relatively lower frequency comparing with other cells. At the same time, we noticed that large number of initial neutrophils could not be reprogrammed back by the chemical cocktail treatment. Comparison between the neutrophils able to be reprogrammed and the cells unable to be reprogrammed showed that the latter one highly expressed microtubules-related genes and cell proliferating genes (Additional file 4: Figure S2C), which might contribute to the reprogramming failure.

To verify the gradual transition of neutrophils from differentiated stage into dedifferentiated stage, we analyzed the cell surface markers on different timepoints after chemical cocktail induction. Comparing with control group, cell percentage of both (Lin,CD115,Siglec-F)^−^Gr1^+^CD11b^+^CXCR4^hi^c-Kit^int^CXCR2^−^ preneutrophils and (Lin,CD115,Siglec-F)^−^Gr1^+^CD11b^+^CXCR4^lo^c-Kit^lo^CXCR2^−^ immature neutrophils was significantly higher and that of (Lin,CD115,Siglec-F)^−^Gr1^+^CD11b^+^CXCR4^−^c-Kit^−^CXCR2^+^ mature neutrophils was significantly lower in the chemical cocktail group on day 8 (Fig. 2d). From the initial induction day to the 8th day after chemical cocktail induction, cell percentage of premature cells and immature cells gradually became more, while mature cells gradually became less (Fig. 2e; Additional file 4: Figure S2D). Identification of the typical neutrophil morphology by Wright-Giemsa staining on different days confirmed the above results. The cells on initial day mainly were band cells and segmented neutrophils and diameter of the nuclear hollowing gradually decreased and even disappeared as time went on after chemical cocktail induction. Cells with high nucleus/cytoplasm ratio were observed obviously on day 6 (Fig. 2f). This observation was also consistent with analyzing the expression of another surface markers together, which defines neutrophil maturation [14]. The data showed that cell percentage of c-kit^hi^/Gr1^neg^ myeloblasts and c-kit^int^/Gr1^neg^ promyelocytes gradually increased from 0.3% to 3.15% and from 7.03% to 36.9% individually. This was paralleled with that of c-kit^neg^/Gr1^hi^ band cells and segmented neutrophils gradually decreased from 65.4% to 11%, from day 2 to day 8 after chemical cocktail induction (Additional file 4: Figure S2E).

In summary, we established a genetic tracing system of the hematopoietic cell fate change based on the cutting-edge artificial barcode labelling and constructed a trajectory map of the chemical cocktail-induced neutrophil reprogramming precession (Fig. 2g), combining with single-cell RNA sequence. The two techniques together are proved to be a powerful tool again for analyzing sophisticated network of cell fate change of both hematopoietic cells and non-hematopoietic cells. In our previous report, we only observed the phenomenon that neutrophils could be reprogrammed into HSPC-like stage, without knowing the precise conversion route and identification of the exact cell type of the induced cells. In this work, we focused on mature neutrophils and found that the cells could be reprogrammed back not only stage by stage into more dedifferentiated status, which was complete reversion of canonical hematopoietic lineage differentiation, but also could skip one or more middle stages. The terminal-induced cells were found to be MPP, which acquired multiple differentiation potential both in vitro and in vivo. Most importantly, these cells could differentiate into lymphoid cells, including T cells and B cells. Thus, the trajectory of chemical cocktail-induced neutrophil reprogramming into MMP is through multiple intermediate stages directly or indirectly. This result not only helps providing new clue for further generating functional hematopoietic stem cells via hematopoietic cell reprogramming, but also helps comprehensively understanding the mechanism of chemical compounds-enabled cell reprogramming of other somatic cells. It is interesting to investigate whether neutrophil reprogramming happens or not in vivo under pathological condition or malignant situation in future, which might provide a novel model for studying hematopoietic regeneration and leukemia development.

## Experimental section

### Mice and derivation of mouse bone marrow cells

Bone marrow cells were derived from 8-week C57BL/6 mouse. After cervical dislocation, whole bone marrow cells were immediately flushed out from long bones (femora and tibia) into 2% fetal bovine serum in phosphate buffered saline. Erythrocytes were removed by 10 min treatment of red blood cell lysis buffer. CD45.2 mice were used as recipients for transplantation. All animal experiments were approved by the Laboratory Animal Resource Center of Ruijin Hospital Affiliated to Shanghai Jiao Tong University School of Medicine.

### Flow cytometry and FACS

Multicolor analysis of neutrophil-derived cells was performed on BD LSKFortessa X-20. The following combinations of cell surface markers were used to define neutrophil populations. PreNeutrophil: (Lin,CD115,Siglec-F)^−^Gr1^+^CD11b^+^CXCR4^hi^c-Kit^int^ CXCR2^−^; immature neutrophil: (Lin,CD115,Siglec-F)^−^Gr1^+^CD11b^+^CXCR4^lo^c-Kit^lo^CXCR2^−^; Mature neutrophil: (Lin,CD115,Siglec-F)^−^Gr1^+^CD11b^+^CXCR4^−^c-Kit^−^CXCR2^+^. Antibodies are listed as follows: Biotin anti-mouse CD115 (CSF-1R) Antibody (Biolegend), Biotin anti-mouse CD170 (Siglec-F) Antibody (Biolegend), Streptavidin-APC-CY7(BD), PE anti-mouse CD117 (c-Kit) Antibody(BD), PE/Cyanine7 anti-mouse CD182 (CXCR2) Antibody (Biolegend), Brilliant Violet 421™ anti-mouse CD184 (CXCR4) Antibody (Biolegend), FITC anti-mouse Ly-6G/Ly-6C (Gr1) Antibody (Biolegend), APC-anti-mouse CD11b Antibody (BD). Flow cytometry data were analyzed by Flowjo 10.4.

### Lentivirus production

Viral particles were produced by transfecting 293T cells with pSMAL-CellTag construct V1 along with packaging plasmid psPAX2 and pMD2G. 293T cells were transfected by jetPRIME® transfection reagent following manufacturer’s instructions. Virus was collected 48 h after transfection and immediately filtered through a low-protein binding 0.45-μm filter.

### CellTagging methodology and single-cell sequencing

Nucleated whole bone marrow cells were first infected with CellTag version 1 lentivirus particles for 16 h and switched to M5300 medium for 8 h. To increase infection efficiency, cells were infected for another 16 h and switched to M5300 medium. Cells were allowed to proliferate for 32 h before splitting into 4 portions. GFP positive fractions were sorted through FACS from 3 portions and immediately taken for single-cell sequencing as day 1. One portion was re-plated in medium supplemented with chemical cocktail and cultured for 7 days. All cells at terminal point were FACS sorted for GFP positive cells then immediately taken for single-cell sequencing.

### Chemical cocktail induction of neutrophils

Freshly isolated neutrophils were cultured in M5300 medium supplemented with murine SCF (100 ng/mL), mFlt3L (100 ng/mL), mIL-3 (50 ng/mL), mIL-6 (50 ng/mL), 1 × 10^−6^ M Hydrocortisone and 1% penicillin–streptomycin solution at the density of 5 × 10^5^ mL^−1^ for the first 6 h. The cells were then cultured in medium supplemented with 0.5 mM VPA (Sigma), 3 μM CHIR99021 (Selleck), and 1 μM Repsox (Selleck) for 7 days, changing medium every 48 h. All cytokines were purchased from Peprotech and were mouse recombinant.

### Transplantation assay

Neutrophils reprogrammed by the chemical cocktail for 7 days from CD45.1 mice were transplanted in 200 μl of DMEM (Gibco) along with 2 × 10^5^ CD45.2 whole bone marrow competitor cells through tail vein injection into γ-irradiated CD45.2 recipient mice (4.5 + 4.5 Gy, with a 4-h interval). Donor cell engraftment efficiency was detected by flow cytometry every 2 weeks after transplantation. Antibodies against B220, CD3, Mac1, Gr1, CD45.1, and CD45.2 were used for peripheral blood analysis of recipient mice.

### Lymphoid differentiation

Bone marrow stromal cell line OP9 and OP9-DL1 were cultured in αMEM (Gibco) supplemented with 20% FBS (Gibco) and 1% penicillin–streptomycin solution. This medium, termed as OP9 medium, was used throughout lymphoid differentiation. On day 0, neutrophils reprogrammed by chemical cocktail for 7 days were plated onto a confluent monolayer of OP9 or OP9-DL cells at a density of 5 × 10^5^ mL^−1^. Cells were cultured in OP9 medium supplemented with mIL-7 (20 ng/mL), mFLT3L (20 ng/mL), VPA (0.5 mM), CHIR99021 (3 μM), and Repsox (1 μM) or OP9 medium supplemented with only cytokines for 7 days. Medium were changed every other day. The co-cultures were trypsinized on day 7 and reseeded onto a fresh confluent OP9 or OP9-DL1 layers in 10 cm dish. Cells were cultured in the same medium for another 7 days for further analysis.

### Single-Cell RNA sequence and analysis

Cells collected before and after chemical cocktail reprogram were detected on the Chromium system (10 × Genomics) individually. The reads of single-cell sequencing FASTQ files were aligned to mouse reference dataset (mm10) by Cell Ranger (v3.1) provided by 10 × Genomics to generate feature-barcode matrixes. The 8-bp random CellTags inserted in cells were calculated on the Shanghai Jiao Tong University HPC.

The single-cell sequence feature-barcode matrixes were analyzed by R-package Seurat (v 3.1.5) on R studio platform following the workflow on the SATIJA LAB. Each Seurat object obtained about 12,000 ~ 13,000 single cells. After filtering out the cells which were not inserted by the CellTag, there are 12,521 and 13,547 cells on day 1 and day 7, respectively. The top 2000 highly variable genes were chosen and the Seurat object data was scaled. The filtered, normalized and scaled Seurat objects were calculated to perform principal component analysis (PCA). Dimension of reduction to compute nearest neighbor graph used the first 20 principle components and resolution to topological divide cells into different clusters was adjusted to 0.5. To illustrate the definite trend, PC 1 and PC 3 were chosen to show the process of reprogramming. The t-distributed Stochastic Neighbor Embedding (t-SNE) visualization was performed to run non-linear dimensional reduction of the Seurat object. Differential expressed genes (DEGs) were calculated by cluster and played a significant role in the following analysis such as cell type identification. The threshold of DEGs was set with adjusted significant expressed value (p_val_adj) ≤ 0.01 and average log fold change (avg_logFC) ≥ 1 to show the different state by volcano plot.

Scanpy, an analysis toolkit for single-cell sequence in python, was used for reconstructing the differentiation trajectory. The preprocessed feature-barcode matrix of neutrophil derived cells was transferred into loom format to reconstruct the trajectory in Scanpy. The mean value of highly variable genes varied from 0.0125 to 3. PCA was computed based on the chosen variable genes. After pre-processing and PCA, the cells were clustered by Leiden algorithm and the neighborhood graph was embedded in 2 dimensions using Factor Analysis (FA). After the relationship of the cluster was constructed by partition-based graph abstraction (PAGA) algorithm, the FA visualization and the PAGA visualization were recomputed to embed for the reprogram trajectory. Resolution of PAGA to topological divide cells into different clusters was adjusted to 1. Feature plots of representative differential expressed genes were used to define the cell type.

## Supplementary information

**Additional file 1**. **Figure S1**: Single-cell RNA sequencing analysis of CellTags-labelledneutrophils under chemical cocktail induction. (A) Distribution ofconfidently mapped reads per cell on day 1 and day 7. (B) Representativegene expression related with HSPC program on day 7. (C) CellTagfrequency in each cell on day 1 and day 7. (D) Cell number with the sameCellTags on both timepoints. (E) Induced cells with HSPC program on day7 are heterogeneous. (F) Dot plot analysis of signature genes with the three gene sets-related phenotypes shown in Figure 1D.

**Additional file 2**. **Table S1.** CellTags in neutrophils.

**Additional file 3**.** Table S2.** Gene list of heatmap in Fig. 1d.

**Additional file 4**. **Figure S2**: Analysis of cell types on different timepoints after chemical cocktail-induced neutrophil reprogramming. (A) Analysis of induced cell types and reprogramming procession from neutrophils based on monocle algorithm. (B) Distribution of CellTags identified in both initial neutrophils on day 1 and induced cells on day 7. Each horizontal line stands for a unique CellTag. (C) Volcano plots represent DEGs in neutrophils unable to be reprogrammed (upper) and able to be reprogrammed under chemical cocktail induction (lower). (D) FACS analysis of CD11b, Gr1, c-Kit, CXCR4, Ly6G, and CXCR2 from day 2 to day 6. (E) FACS analysis of c-Kit and Gr1 from day 2 to day 8.

## Data Availability

The datasets used and/or analyzed during the current study are available from the corresponding author on reasonable request.

## Abbreviations

HSPC: Hematopoietic stem/progenitor cell
GFP: Green fluorescent protein
VPA: Valproic acid
HSC: Hematopoietic stem cells
MPP: Multipotent progenitors
GMP: Granulocyte/monocyte progenitors
CMP: Common myeloid progenitors
MEP: Megakaryocyte/erythrocyte progenitors
PAGA: Partition-based graph abstraction
t-SNE: T-distributed Stochastic Neighbor Embedding.
