# Supplementary figures and images for: Trajectory of chemical cocktail-induced neutrophil reprogramming
Source: J Hematol Oncol. 2020 Dec 10;13:171. doi: 10.1186/s13045-020-01008-8 (PMC7727137; doi:10.1186/s13045-020-01008-8)

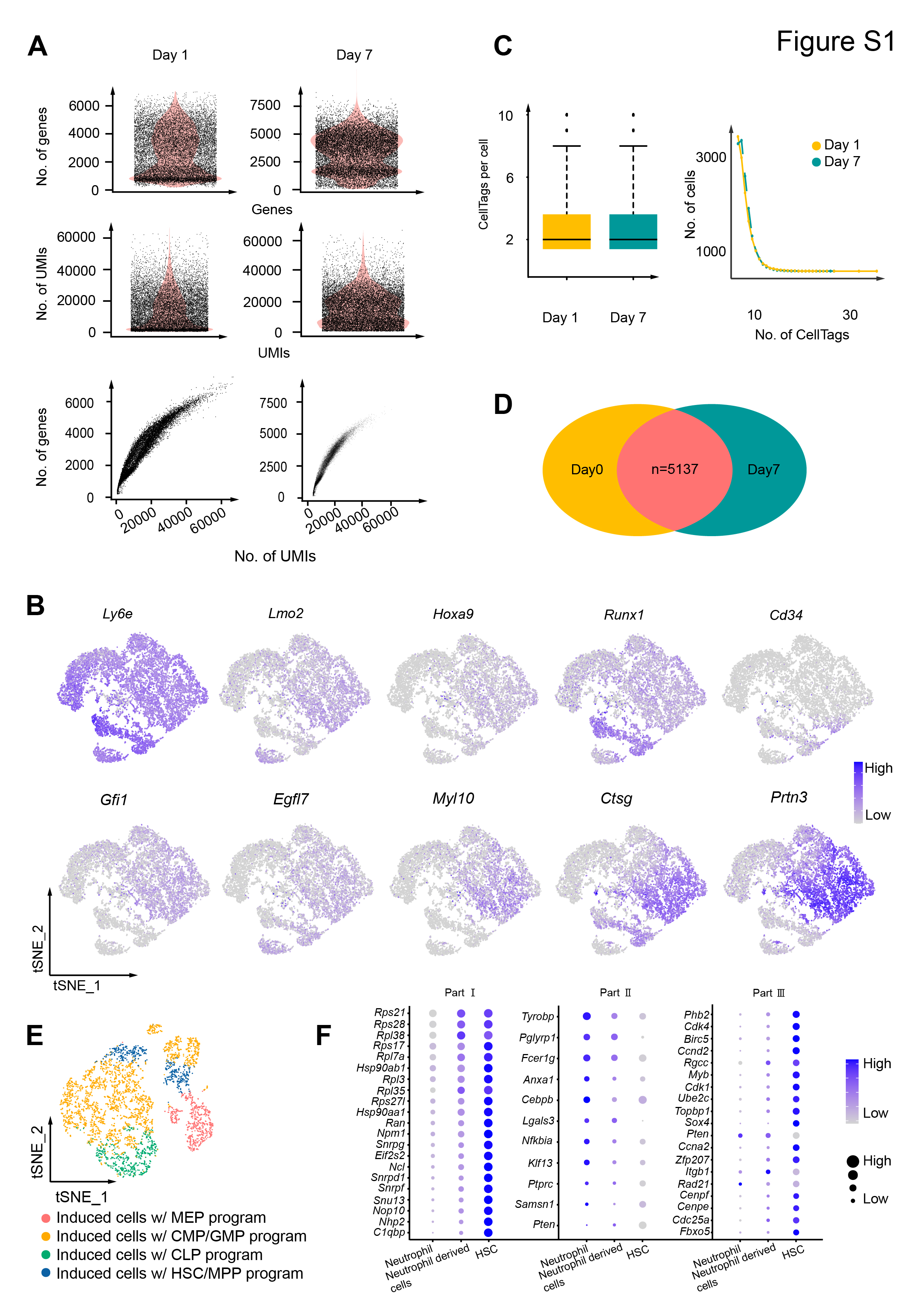

Supplement: Supplementary file 1 — Additional file 1. Figure S1: Single-cell RNA sequencing analysis of CellTags-labelledneutrophils under chemical cocktail induction. (A) Distribution ofconfidently mapped reads per cell on day 1 and day 7. (B) Representativegene expression related with HSPC program on day 7. (C) CellTagfrequency in each cell on day 1 and day 7. (D) Cell number with the sameCellTags on both timepoints. (E) Induced cells with HSPC program on day7 are heterogeneous. (F) Dot plot analysis of signature genes with the three gene sets-related phenotypes shown in Figure 1D. [file 13045_2020_1008_MOESM1_ESM.jpg]

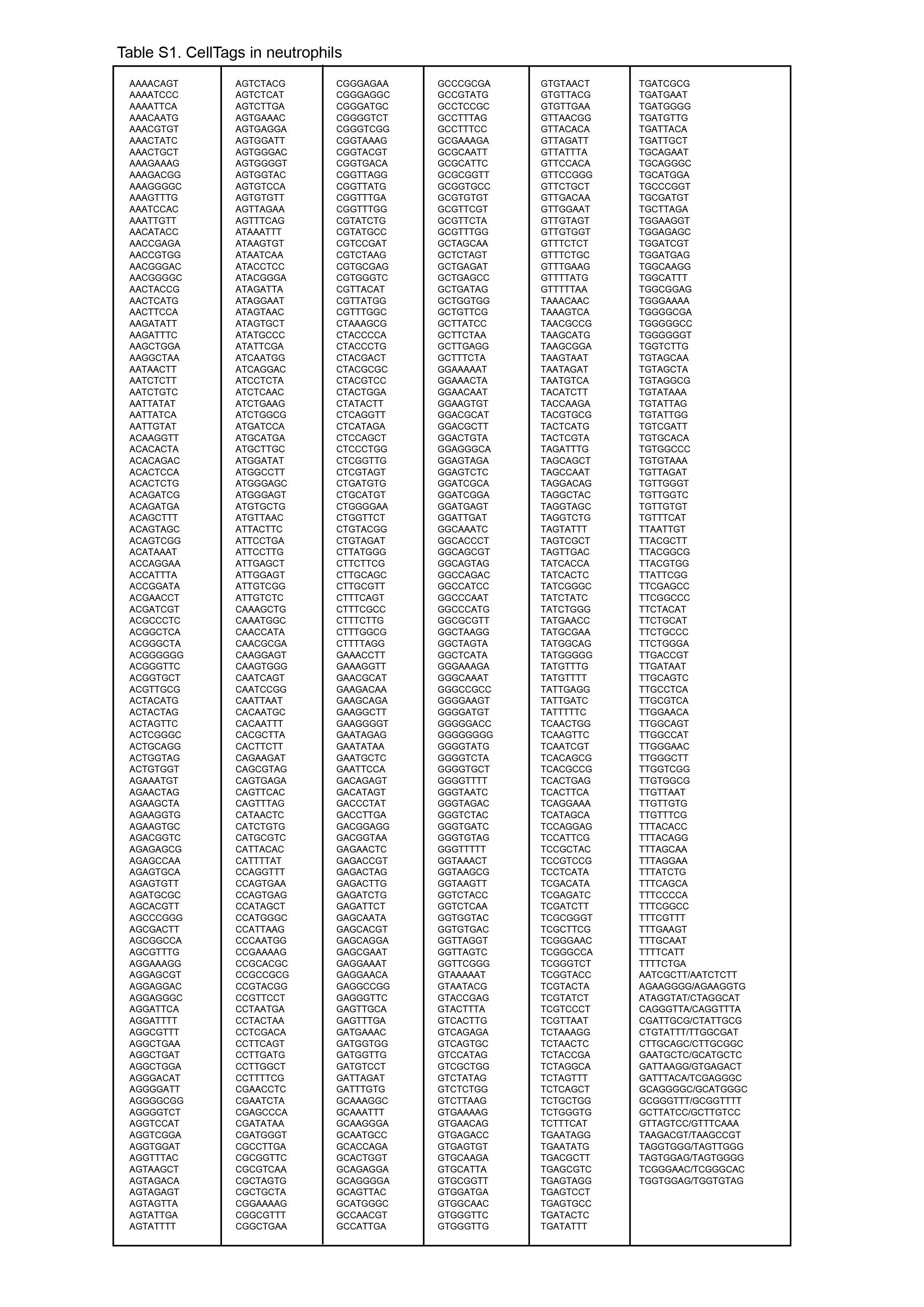

Supplement: Supplementary file 2 — Additional file 2. Table S1. CellTags in neutrophils. [file 13045_2020_1008_MOESM2_ESM.jpg]

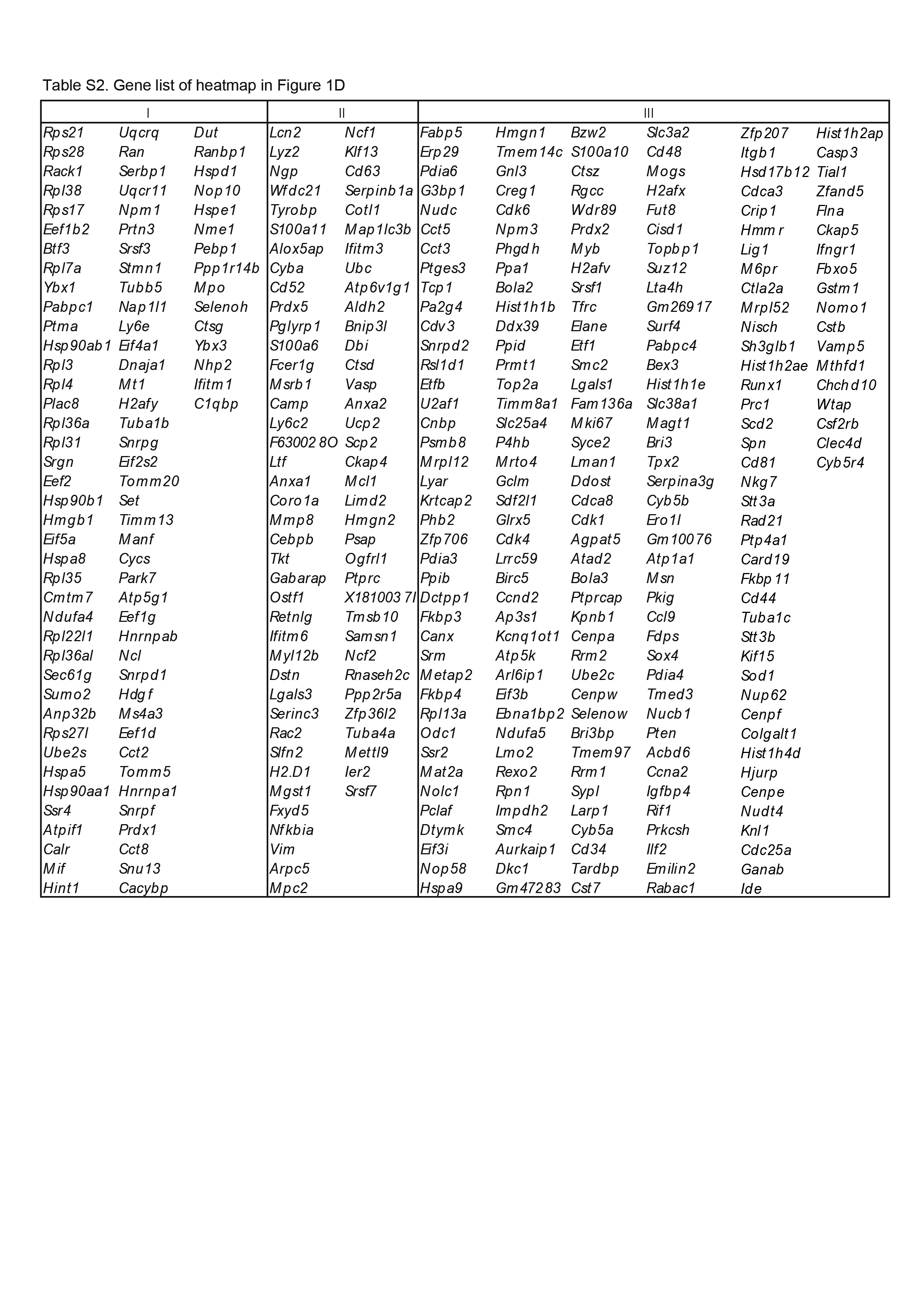

Supplement: Supplementary file 3 — Additional file 3. Table S2. Gene list of heatmap in Fig. 1d. [file 13045_2020_1008_MOESM3_ESM.jpg]

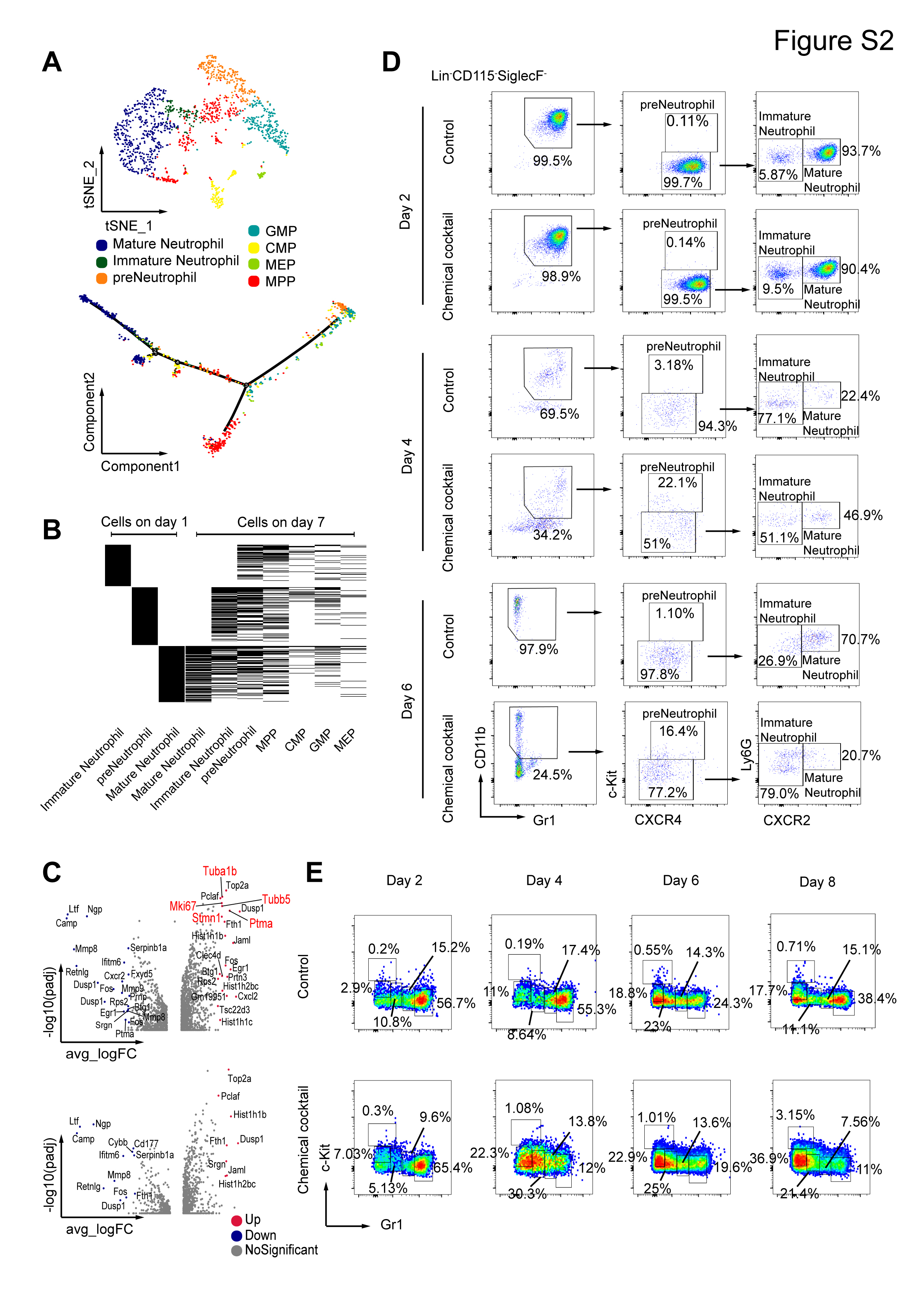

Supplement: Supplementary file 4 — Additional file 4. Figure S2: Analysis of cell types on different timepoints after chemical cocktail-induced neutrophil reprogramming. (A) Analysis of induced cell types and reprogramming procession from neutrophils based on monocle algorithm. (B) Distribution of CellTags identified in both initial neutrophils on day 1 and induced cells on day 7. Each horizontal line stands for a unique CellTag. (C) Volcano plots represent DEGs in neutrophils unable to be reprogrammed (upper) and able to be reprogrammed under chemical cocktail induction (lower). (D) FACS analysis of CD11b, Gr1, c-Kit, CXCR4, Ly6G, and CXCR2 from day 2 to day 6. (E) FACS analysis of c-Kit and Gr1 from day 2 to day 8. [file 13045_2020_1008_MOESM4_ESM.jpg]
